# Genetic Alterations in Poorly Differentiated and Undifferentiated Thyroid Carcinomas

**DOI:** 10.2174/138920211798120853

**Published:** 2011-12

**Authors:** Paula Soares, Jorge Lima, Ana Preto, Patricia Castro, João Vinagre, Ricardo Celestino, Joana P Couto, Hugo Prazeres, Catarina Eloy, Valdemar Máximo, M Sobrinho-Simões

**Affiliations:** 1 Institute of Pathology and Molecular Immunology, University of Porto (IPATIMUP), 4200-465 Porto, Portugal; 2 Medical Faculty, University of Porto, 4200-465 Porto, Portugal; 3 CBMA (Centre of Molecular and Environmental Biology)/Department of Biology, University of Minho, Campus de Gualtar, 4710-057 Braga, Portugal; 4 Institute of Biomedical Sciences Abel Salazar (ICBAS), University of Porto, 4099-003 Porto, Portugal; 5 Department of Pathology, Hospital São João, 4200-465 Porto, Portugal; 6 Laboratory of Molecular Pathology of the Portuguese Institute of Oncology of Coimbra, EPE, 3000-075 Coimbra, Portugal

**Keywords:** Poorly differentiated thyroid carcinoma, undifferentiated thyroid carcinoma, BRAF, RAS, P53, β-catenin, PI3K, telomerase.

## Abstract

Thyroid gland presents a wide spectrum of tumours derived from follicular cells that range from well differentiated, papillary and follicular carcinoma (PTC and FTC, respectively), usually carrying a good prognosis, to the clinically aggressive, poorly differentiated (PDTC) and undifferentiated thyroid carcinoma (UTC).

It is usually accepted that PDTC and UTC occur either *de novo* or progress from a pre-existing well differentiated carcinoma through a multistep process of genetic and epigenetic changes that lead to clonal expansion and neoplastic development. Mutations and epigenetic alterations in PDTC and UTC are far from being totally clarified. Assuming that PDTC and UTC may derive from well differentiated thyroid carcinomas (WDTC), it is expected that some PDTC and UTC would harbour genetic alterations that are typical of PTC and FTC**.** This is the case for some molecular markers (BRAF and NRAS) that are present in WDTC, PDTC and UTC. Other genes, namely P53, are almost exclusively detected in less differentiated and undifferentiated thyroid tumours, supporting a diagnosis of PDTC or, much more often, UTC. Thyroid-specific rearrangements RET/PTC and PAX8/PPARγ, on the other hand, are rarely found in PDTC and UTC, suggesting that these genetic alterations do not predispose cells to dedifferentiation. In the present review we have summarized the molecular changes associated with the two most aggressive types of thyroid cancer.

## INTRODUCTION

Undifferentiated thyroid carcinoma (UTC), a term we will use as a synonym for anaplastic carcinoma, ranks among the most lethal of all human malignancies [1, 2] and represents nowadays less than 3% of all clinically recognized malignant thyroid neoplasms [2]. UTC is more common in females and in elderly patients, usually in their seventh decade. UTCs are usually non-encapsulated, extensively invading the perithyroid tissues. Grossly, the neoplasms have fleshy, tan-white appearance, with haemorrhagic and necrotic areas, and are composed, at the microscopic level, of cells with spindle, polygonal or giant-cell morphology. Due to airway obstruction, local invasion, vascular invasion and/or distant metastases to lung and bone, and to a striking resistance to any type of therapy, the patients usually die within a few months after diagnosis [3].

UTCs may coexist with, or follow, either a well differentiated thyroid carcinoma (WDTC) or a long standing goiter in about 30% of cases [1, 4]. The anaplastic transformation of a WDTC, namely a papillary thyroid carcinoma (PTC) or, less often, a follicular thyroid carcinoma (FTC) or a poorly differentiated thyroid carcinoma (PDTC), is accepted as an intermediate step of UTC development [4]. The absence of foci of WDTC close to UTC in many cases may be explained by insufficient sampling or by overgrowth of the UTC, obscuring a putative pre-existing WDTC [1]. Furthermore, there are UTCs which are thought to appear *de novo*. Although some authors have reported the presence of calcitonin immunostaining of neoplastic cells in some cases of UTC, the existence of a UTC made of C-cells is not recognized [2], since the origin of UTC from C-cells has never been demonstrated. 

The diagnostic situation regarding PDTC is much less clear than that of UTC. The diagnostic criteria applied to PDTC by different groups over the last 25 years have varied a lot and the first consensus was only obtained in the 2004 WHO classification [2]. PDTC was then recognized as a distinct entity, being defined as a thyroglobulin-producing non-follicular non-papillary thyroid carcinoma, disclosing distinctive high grade features (necrosis and mitoses) and a well-developed insular, trabecular or solid pattern of growth present in the majority of the tumour, quite often admixed altogether within the same tumour and less frequently in a “pure” form. This definition places PDTC as a follicular cell-derived carcinoma with morphological and biological attributes intermediate between differentiated and undifferentiated thyroid carcinomas [2]. PDTC represents 2-4% of all thyroid carcinomas and comprises a morphologically heterogeneous group of tumours [1, 5-7]. 

PDTC encompasses at least two major types of tumours: the so-called insular or insular-like carcinomas and a heterogeneous group of carcinomas that also fit into the umbrella descriptive designation of PDTC displaying predominantly trabecular or solid growth pattern [8]. Using immunohistochemistry, lectin histochemistry, electron microscopy and molecular genetics, we verified that the “pure” insular or insular-like PDTC resemble follicular carcinomas (namely widely invasive follicular carcinomas with a solid growth pattern), whereas in a proportion of PDTC displaying trabecular or solid growth pattern one can find features characteristic of PTC [8]. In both instances, foci of necrosis and high mitotic rate may be used in individual cases as signs suggestive of PDTC, but should not be considered as absolute criteria, namely whenever dealing with a solid/trabecular variant of PTC, in which the presence of necrotic foci and/or numerous mitoses should not lead to a diagnosis of PDTC, provided the nuclei are of the PTC-type throughout the tumour [7, 9]. 

At present there is a general consensus to diagnose a PDTC on the following issues: a) Presence of conventional criteria of malignancy and unequivocal signs of follicular cell derivation of tumour cells; b) Presence of a solid, trabecular, or insular pattern of growth in an otherwise malignant thyroid tumour; c) Absence of the conventional nuclear features of PTC; d) Presence of convoluted nuclei and/or mitotic activity (>3 mitoses/10 HPF) and/or necrosis; e) PDTCs composed of oxyphilic, mitochondrion-rich Hürthle cells do not constitute a separate entity but rather an oncocytic variant of PDTC; and f) The finding of a concurrent better differentiated papillary or follicular carcinoma component does not preclude the diagnosis of PDTC [7, 10].

Genetic alterations in PDTC and UTC are far from being totally clarified. The quite different percentages of molecular alterations reported in different series of PDTC (e.g. BRAF mutations, RET/PTC rearrangement and β-catenin mutations), probably reflect the discrepancies in the classification and the heterogeneity of tumours that are often included under the umbrella category of PDTC (namely PTC with morphological signs of poor differentiation: solid, insular and trabecular foci). Assuming that PDTC and UTC may derive from WDTC, it is expected that some PDTC and UTC will harbour genetic alterations that are typical of PTC and FTC [11]. As we will see, this is the case for some molecular markers (BRAF and NRAS) that are present in WDTC, PDTC and UTC. Other genes, namely P53, are almost exclusively found in PDTC and UTC, indicating a UTC or, less often, a PDTC diagnosis. Thyroid specific rearrangements namely RET/PTC and PAX8/PPARγ, on the other hand, are rarely found in PDTC and UTC, suggesting that these genetic alterations do not predispose cells to dedifferentiation.

## BRAF IN POORLY DIFFERENTIATED AND UNDIFFERENTIATED THYROID CARCINOMAS

The RAF family encodes serine/threonine kinases that are key signal transducers of diverse extracellular stimuli and activate mitogen-activated protein kinase (MAPK) signalling pathway [12]. The RAF family is composed of three different isoforms, ARAF, RAF- 1 (or CRAF) and BRAF that, despite the high sequence and structural similarities, are differentially regulated. After the identification of BRAF mutations in a wide variety of human cancers, namely melanoma, ovarian carcinoma and microsatellite unstable colorectal carcinomas [13], we and others have reported a high prevalence (30% to 69%) of BRAF point mutations in PTC [14-18]. These mutations were roughly limited to the hotspot identified in melanomas, T1799A leading to V600E substitution. Other, less frequent, BRAF mutations were also detected in PTC, namely the K601E that we found almost exclusively in the follicular variant of PTC [19].

BRAF mutations in PTC seem to be an alternative event to RET/PTC rearrangements and to RAS mutations [16, 18]. There is also recent evidence showing an association between the presence of BRAF mutations and PTC recurrence, mortality and resistance to radioiodine therapy [20-22], that is probably related to the association of BRAF mutations with silencing of iodine-handling genes [23] and to the demonstration that BRAF-mutated PTC may evolve to PDTC and UTC.

In accordance with a stepwise progression model, BRAF mutations are more frequent in PDTC arising from PTC, than in PDTC associated with FTC [14, 24]. This was clear when we evaluated a group of PDTC exclusively composed of insular and insular-like tumours, thus excluding PDTC with foci displaying PTC nuclei. No BRAF mutations were detected in this group, supporting the assumption that pure insular and insular-like PDTC are more closely related to FTC than to PTC [24]. BRAF mutations were nevertheless described in PDTC with PTC-like nuclei, as well as in PDTC coexisting with foci of PTC [14, 22].

Treatment of thyroid carcinomas, including PTC, FTC and PDTC, is usually achieved through surgical removal and the use of radioactive iodine (^131^I). Although the large majority of WDTC respond well to radioiodine therapy, there are thyroid tumours not responsive to this therapy, namely some PTC variants that are inoperable and have lost radioactive iodine avidity, as well as some carcinomas composed of oncocytic (Hürthle) cells and some less differentiated thyroid tumours [22]. The demonstration that BRAF mutations are associated with silencing of iodine-transporting genes in thyroid cancer indicates that targeting BRAF may contribute for improving the iodine uptake ability of thyroid cancer cells [21, 23, 25]. 

Ricarte-Filho *et al. *[22] reported recently that PDTC, FDG-PET positive tumours refractory to radioactive iodine treatment, harbour BRAF mutations in 39% of the samples, whereas non-refractory PDTC display a significantly lower frequency of BRAF mutations (12%) [22].

BRAF mutations have also been described in 10% to 44% of UTC [14, 22, 24]; in such BRAF-mutated UTC, the mutations are frequently detected in adjacent foci of PTC that are thought to represent the origin of UTC. This supports the idea that BRAF mutations can be implicated in the progression of WDTC to PDTC or UTC [26, 27], at variance with what has been described for other genetic changes, namely RET/PTC and PAX8/PPARγ rearrang-ements (see below). 

## RAS IN POORLY DIFFERENTIATED AND UNDIFFERENTIATED THYROID CARCINOMAS

RAS genes, namely N-RAS but also H-RAS and K-RAS, are consistently found mutated in less differentiated thyroid tumours. The prevalence of RAS mutations ranges between 18 and 55% in PDTC [9] and between 4 and 60% in UTC [22, 28-32]. 

The prognostic value of RAS mutations in thyroid cancer is not well established. In some series, RAS mutations were shown to be associated with aggressive tumour phenotypes and poor prognosis [9, 30], whereas in others such an association was not observed [22]. It has been advanced that cases with mutated N-RAS are significantly associated with the appearance of haematogenous (particularly bone) metastases, suggesting a role of RAS genes activation in the metastatic capability of these tumours [28, 29, 33]. 

Due to the association found between RAS mutations and guarded prognosis in PDTC and UTC, Wang *et al. *proposed that a particular attention should also be paid to WDTC, namely FTC, harbouring RAS mutations [34, 35].

## P53 IN POORLY DIFFERENTIATED AND UNDIFFERENTIATED THYROID CARCINOMAS

It was hypothesized that UTC may progress from BRAF-mutated PTC by acquisition of an additional p53 mutation [32]. Protein expression and gene mutation analysis of p53 in WDTC show that p53 mutation is an extremely rare event in these carcinomas; in fact, more than 98% of the WDTC (PTC and FCT) analysed have a normal p53 gene [11, 36-40].

In this respect, thyroid tumours do not follow the classical Vogelstein model, in which p53 mutation is a crucial step in the first phases of progression (from adenoma to carcinoma) [41]. In thyroid tumours, p53 gene inactivation seems to play an important role in the progression from differentiated to undifferentiated carcinoma, being a late event in the carcinogenic process and occurring together with a marked increase of cell proliferation [42-45]. The analysis of the expression and/or mutations of p53 in PTC co-existing with undifferentiated carcinomas, has shown that p53 expression/mutation is only found in the undifferentiated components [45]. 

P53 mutations have been reported in approximately 26% of PDTC and in more than 60% of UTC [11]. Virtually all the mutations reported are located in the known hot-spots (exons 5-9), being 273 the codon most often affected [37-39, 46, 47]. 

Progression and dedifferentiation in thyroid tumours seems to follow a particular pattern. It is possible that the progression *in vivo *results from a two-step mechanism, in which there is the need for a differentiation switch, but also for a p53 mutation to arise independently in the same cell, before any selective advantage is obtained [48, 49]. Taking in consideration the data on the progression of WDTC (no p53 mutations) to PDTC and UTC, which frequently harbour p53 inactivation, gross genetic alterations and aneuploidy, it is likely that in the more advanced steps of progression, p53 inactivation (“suppressor pathway”) represents the most frequent mechanism of chromosome instability in thyroid cancer. This is in accordance with the sequential increase in chromosomal complexity observed in CGH studies from WDTC to PDTC and UTC, in terms of the presence and number/case of CGH detectable abnormalities [50-53].

The frequency of p53 mutations in UTC reported in the different series lies between 60 and 83% [11, 22, 38-39]. Even considering the different sensitivities of the different methodologies, it is tempting to advance that, at least in a minority of cases, alterations in genes other than p53 can lead to a similar end stage of thyroid tumour development. The prognosis is similar in UTC with and without p53 mutations, a finding that supports the existence of alternative pathways to achieve a similar end result; WNT pathway and CDK/CDKI molecules, which were found altered in thyroid cancer cell lines and/or primary tumours [54, 55] appear to be good candidates (see below). We have also evaluated the role of p63 that, in its deltaN form, can act as a dominant negative of p53, in dedifferentiation/progression from WDTC to UTC. Our results do not support the hypothesis that p63 overexpression may serve as an alternative molecular mechanism to overcome p53 activity in WDTC or in UTC [56].****

## RET/PTC AND PAX8/PPARγ REARRANGEMENTS IN PDTC AND UTC

Rearrangements involving the RET oncogene (RET/PTC) are commonly found in PTC. RET/PTC rearrangements lead to a constitutively expressed and activated form of the RET proto-oncogene that is able to trigger transformation in PTC. The frequency of RET/PTC in PDTC has been reported to be considerably lower than in PTC (13-17%) [22, 57] or absent [9, 58]. Moreover, RET/PTC-positive PDTC are not associated with increased aggressiveness or poor patient survival and usually show histological evidence indicating coexistence with or possible evolution from a PTC often diagnosed as a classic, solid or tall cell variant PTC [7, 22, 57]. Concerning UTC, all previous studies reported an absence of RET/PTC rearrangements in this setting [22, 58]; only the study by Mochizuki *et al. *[59], who studied seven composite UTC (UTC having a PTC component) and 14 single component UTC, has found the presence of a RET/PTC3 rearrangement in both components (UTC and PTC) of one composite UTC, whereas all 14 single component UTC were RET/PTC-negative. 

The aforementioned works suggest that RET/PTC rearrangements do not seem to play a role in the progression of WDTC to PDTC. This appears also to be case for PAX8/PPARγ rearrangements, that were originally discovered in FTC and, later on, in follicular adenomas and follicular variant of PTC [60-63]; PAX8/PPARγ rearrangements are absent from all PDTC and UTC so far analysed [9, 22].

## WNT PATHWAY

β-catenin mutations were reported in 25% and 65% of PDTC and UTC, respectively [55, 64, 65]. The activating mutations cluster in exon-3, at the phosphorylation sites for ubiquitination and degradation of β-catenin, and are associated with aberrant nuclear immunoreactivity, which is consistent with Wnt pathway activation [64, 65]. According to these studies, β-catenin mutations are restricted to PDTC and UTC, being significantly associated with poor prognosis and tumour differentiation, but not with conventional prognostic indicators for thyroid cancer. The results from Kim *et al. *[66], showing that transient overexpression of Wnt-1 or β-catenin in FRTL-5 cells decreased thyroperoxidase (TPO) mRNA, and suppressed TPO-promoter activity, corroborate a putative role for β-catenin activation in loss of differentiation of thyroid cancer cells. 

In contrast to the aforementioned results, Rocha *et al. *[67] did not find mutations in β-catenin or E-cadherin genes in a series of PDTC; however, there was altered expression of E-cadherin/β-catenin at the protein level, suggesting that loss of E-cadherin rather than β-catenin mutations is the crucial event in determining the differentiation 'level' of thyroid carcinomas [67]. The quite different percentages of β-catenin mutations reported in different series of PDTC [64, 65, 67, 68] probably reflect the heterogeneity of tumours that are often included under the umbrella category of PDTC [8]. In other series of UTC, nuclear and cytoplasmic positivity for β-catenin was found in 40% of the cases but mutations were only detected in 4.5% of the cases [68]. The same authors reported mutations in adenomatous polyposis coli (APC) and Axin 1 in 9.0%, and 81.8% of the ATC samples, respectively [68]. 

## TELOMERASE: LIMITLESS OF PROLIFERATIVE POTENTIAL?

Telomerase activation is known to be a hallmark of cancer [69], being detected in 80 to 90% of malignant tumours [70, 71]. Some tumours may maintain their telomeres by an alternative mechanism, telomerase-independent, designated as ALT (alternative lengthening of telomeres), which appears to maintain telomeres through recombination based interchromosomal exchange of sequence information [72, 73].

Thyroid tissue is a conditional-renewal tissue, which proliferates very slowly and rarely; human thyroid cells are supposed to divide about five times in the adult life [74]. In the thyroid gland there is not a well-defined stem cell population that might constitute a pool of cells responsible for retaining the capacity of division. Some authors, including us, have proposed that the so-called Solid Cell Nests (SCNs) of the thyroid, which are the embryonic remnants of the ultimobranchial body, may represent the pool of thyroid stem cells as they express several stem cell markers, namely telomerase [75]. Despite this, normal thyroid tissue is thought to be telomerase negative thus raising the possibility that reactivation of telomerase may be a useful marker for tumour development and, if there are quantitative differences between benign and malignant lesions, for making the differential diagnosis in difficult cases [76]. 

Several studies have examined telomerase activity in thyroid lesions and surrounding normal tissues using polymerase chain reaction (PCR) based TRAP (Telomeric Repeat Amplification Protocol) assay for detection of telomerase activity and RT-PCR or quantitative real time RT-PCR for detection of hTERT mRNA (Table **1**). The results obtained in all the studies using TRAP to determine telomerase activity lead to a persistent source of uncertainty.

Thyroid carcinomas apparently display less frequent telomerase activation than other human carcinomas, being present in about 66% of all the thyroid carcinomas analysed to date (Table **1**). Telomerase activation seems to be more frequent in UTC being detected in about 78% of the cases analysed (Table **1**); this finding suggests that telomerase may contribute (or be associated) to more aggressive behaviour of thyroid cancer [77-79]. 

Telomerase activation was measured by expression of hTERT gene by RT-PCR in four studies. These studies showed a significant association between hTERT expression and telomerase activity. hTERT expression was not detected in normal adjacent thyroid tissue, but it was found in a high percentage of the carcinomas namely in UTC [77, 78, 80, 81] (Table **1**). Putting together the results obtained in the measurement of telomerase activity by several authors (Table **1**), the presence of telomerase is reported in 48% of PTC and in 71% of FTC (Table **1**).

Summing up, telomerase activation is less frequent in thyroid carcinomas (about 66%) than in other types of human cancer (80 to 90%). The available evidence suggest that telomerase activity is up-regulated in thyroid neoplastic cells and may be a marker of aggressiveness in thyroid tumours, since it has been associated with malignancy, invasiveness, advanced thyroid carcinomas and with the progression of WDTC to UTC [77-79, 82-84]. As far as we are aware there are no studies reporting telomerase activation in PDTC.

## OTHER GENETIC ALTERATIONS

Due to the aggressive nature of most PDTC and every UTC, efforts have been put on the identification of other genetic alterations in less differentiated and undifferentiated thyroid carcinomas that could identify new therapeutic targets. This is the case for tyrosine kinase receptors that lie upstream of the two pathways – MAPK and PI3K/Akt – most commonly activated in UTC. Liu *et al. *[85] searched for alterations (mutations and copy gains) in several tyrosine kinase receptors (EGFR, PDGFRα, PDGRFβ, VEGFR1, VEGRF2, KIT and MET) in a series of UTC and FTC. No mutations were observed in any of these receptors, but the frequency of copy number gain was high, particularly among UTC; overall, it was detected at least one copy number gain in 80% of UTC. Putting together the results on copy number gains and commonly found mutations (BRAF, RAS, PIK3CA), 90% of UTC harboured at least one genetic alteration [85]. Considering the studies reported to date, it is evident that most of the genetic alterations in PDTC and UTC lead to activation of MAPK and PI3K/Akt pathways. This is strengthened by the recent finding of ALK (anaplastic lymphoma kinase) oncogenic mutations in 11% UTC cases; these mutations promote cell focus formation, anchorage-independent growth, and cell invasion and also increase the ability of ALK to activate the PI3K/Akt and MAP kinase pathways in established mouse cells [86]. It was proposed that the presence of ALK mutations in UTC may provide a basis for analyzing the clinical role of ALK inhibitors in this setting [86]. 

We have recently described that UTCs present frameshift mutation and genomic loss of LRP1B gene [87]. LRP1B localizes at 2q21, a susceptibility locus for familial non-medullary thyroid cancer and encodes for a member of the endocytic low-density lipoprotein receptor superfamily. We have found that LRP1B frequently displays genomic loss both in cell lines and also in sporadic thyroid tumours, including 60% of UTCs. UTCs also show frequent methylation of the promoter region and, accordingly, the expression of LRP1B was lost in more than 80% of UTCs [87]. 

There is increasing evidence showing that altered expression of microRNAs plays an important role in cancer development. In this regard, Visone *et al. *[88] analysed the microRNA (miR) profile of UTC in comparison to the normal thyroid, having found that UTC displays an aberrant miR expression profile that clearly differentiates it from normal thyroid tissues. Overall, UTC showed downregulation of the majority of miR, four of them (miR-30d, miR-125b, miR-26a and miR-30a-5p) being downregulated to a 3-fold level, suggesting that the downregulated miRs in UTC can function as tumour suppressor or differentiation genes [88]; these results were further confirmed by Schwertheim *et al. *[89], who found that the same four miRs were significantly upregulated in PTC and downregulated in UTC, whereas in PDTC only 3/5 were downregulated. In another study, Braun *et al. *[90] also showed that UTC presented a general downregulation of miRs comparing to normal thyroid and also to PTC and FTC. The authors identified two significantly decreased microRNA families (miR-200 and miR-30) that unambiguously distinguish UTCs from PTC and FTC. Expression of these microRNAs in mesenchymal UTC-derived cells reduced their invasive potential and induced mesenchymal–epithelial transition, suggesting that the altered microRNA signatures in UTC may play a role in the promotion of de-/transdifferentiation (epithelial-mesenchymal transition) and invasion of these neoplasias [90]. 

Other genes and pathways important for the development of PDTC and UTC may include, for example, those involving metabolic remodelling; in fact, after the recent identification of isocitrate dehydrogenase mutations in glioblastomas (that share the aggressive behaviour with PDTC and UTC), other types of cancer have also been screened for IDH mutations. In thyroid cancer, IDH mutations were detected in 11% of the UTC cases [91]; although IDH mutations do not seem to be particularly prevalent in UTC, they are functionally relevant and may put forward a new therapeutic approach for such a lethal cancer as UTC [91]. 

## Figures and Tables

**Table 1.** Summary of Published Studies on Telomerase Activity in Thyroid Neoplasias

Authors	Normal adjacent thyroid tissue**	Follicular adenoma**	Follicular carcinoma**	Papillary carcinoma**	Anaplastic carcinoma**
Saji *et al.* [92]	0/10	0/3	_	20/30	_
Saji *et al.* [80]*	0/12*	2/7*	6/6*	9/13*	_
Aogi *et al.* [81]	_	0/11	3/3	5/5	1/2
(same cases)	_	5/11*	3/3*	2/5*	2/2*
Aogi *et al.* [93]	_	0/9	3/3	5/5	1/2
Yashima *et al.* [94]	3/22	1/5	0/2	4/11	_
Umbricht *et al. * [83]	0/22	5/23	11/11	_	_
Cheng *et al.* [95]	_	4/14	10/11	12/23	_
Brousset *et al. * [79]	0/20	1/12	4/6	3/15	2/3
De Deken *et al. * [96]	3/28	1/28	_	_	_
Okayasu *et al.* [84]	0/26	9/23	3/4	16/26	_
Haugen *et al.* [97]	3/14	0/14	0/3	10/14	0/1
Lo *et al.* [98]	0/35	0/9	0/2	15/52	1/2
Onoda *et al.* [99]	5/14	0/2	_	9/16	1/1
Kammori *et al.* [100]	1/21	3/9	3/3	7/8	_
Sebesta *et al.* [101]	_	3/4	_	2/3	_
Matthews *et al. * [102]	0/10	3/22	6/16	8/37	_
Takano T *et al. * [78]	_	_	_	_	12/12*+
Total	15/234 (6.4%)	37/212 (17.5%)	52/73 (71.2%)	127/263 (48.3%)	18/23 (78.3%)

* Telomerase subunit hTERT was analysed by RT-PCR *+ Telomerase subunit hTERT was analysed by real-time quantitative RT-PCR; all the other studies were performed only by TRAP.

** Positive cases/total cases (percentage positive in parentheses).
